# Using Spatial Video to Analyze and Map the Water-Fetching Path in Challenging Environments: A Case Study of Dar es Salaam, Tanzania

**DOI:** 10.3390/tropicalmed2020008

**Published:** 2017-04-11

**Authors:** Sarah L. Smiley, Andrew Curtis, Joseph P. Kiwango

**Affiliations:** 1 Department of Geography, Kent State University at Salem, 2491 State Route 45 South, Salem, OH 44460, USA; 2 Department of Geography. Kent State University, 413 McGilvrey Hall, Kent, OH 44242, USA; acurti13@kent.edu; 3 University of Dar es Salaam, P.O. Box 35027, Dar es Salaam, Tanzania; kiwangojoseph@gmail.com

**Keywords:** water fetching, spatial video, Tanzania, water access, informal settlements, micro-geographies

## Abstract

Access to clean drinking water remains a significant health problem in the developing world. Traditional definitions of water access oversimplify the geographic context of water availability, the burden of water collection, and challenges faced along the path, mainly due to a lack of fine scale spatial data. This paper demonstrates how spatial video collected in three informal areas of Dar es Salaam, Tanzania, can be used to quantify aspects of the walk to water. These include impediments encountered along the path such as changes in elevation and proximity to traffic. All are mapped along with classic health-related environmental and social information, such as standing water, drains, and trash. The issue of GPS error was encountered due to the built environment that is typical of informal settlements. The spatial video allowed for the correction of the path to gain a more accurate estimate of time and distance for each walk. The resulting mapped health risks at this fine scale of detail reveal micro-geographies of concern. Spatial video is a useful tool for visualizing and analyzing the challenges of water collection. It also allows for data generated along the walk to become part of both a household and local area risk assessment.

## 1. Introduction

Accessibility to clean water remains a largely elusive goal in many developing world urban areas [1,2,3]. Appropriate and adequate water access, sanitation, and personal hygiene (WASH) are vital in maintaining healthy households. WASH is, however, a spatially complex situation comprised of physical locations (such as water sources, transportation routes, and households), various impediments to access (such as dangerous or unsafe spaces and illegal or unpredictable water rationing), environmental factors (such as changes in elevation and drainage patterns), and social and behavioral impacts [4]. Of concern are the myriad health impacts and pathogens associated with where a household fits into this complexity. For example, is it close to contaminated water or mud, which may result in pathogens leading to different enteric diseases [5,6]? 

Even within an urban area typically regarded as health vulnerable, such as an informal settlement, there will still be a considerable degree of heterogeneity [7,8]. There will be sections of lower elevation where water and mud pool and areas disproportionately affected by the channels that carry flood water and even sewage potentially washed from pit latrines [9]. Furthermore, irregular or absent trash collection can provide suitable habitats for vermin and breeding grounds for mosquitoes [10,11]. As a result, it should be possible to map each household as a function of its proximity to these risks, including areas where settled water and mud persist [12], open sewage channels [13], toilets [4,14], and trash dumps. However, this type of mapping at this scale remains largely unachievable because informal settlements are severely lacking in fine spatial scale data [8,15], which are the type of data that could be used to make effective interventions. Previous studies that have included spatial aspects of WASH sometimes include proximity to risks in and around the home [4]. What is often missed, however, are activity spaces and daily water-fetching paths [16], which are a vital part of the household regime. 

Although 56 percent of Tanzanians have access to an improved source of clean water, only 13 percent have piped-on-premises water; thus, most households must walk to fetch their water [17]. Water access is defined by proximity to a water source, so if a household is within a prescribed distance (usually 1 km) of a source of clean water, then it has water access [18]. However, local conditions will cause considerable variation in the time taken to walk that distance [19]. 

This distance also has a direct impact on the health of those in the household. For example, it has been found in the case of childhood diarrhea in Tanzania that morbidity and mortality significantly worsen with every 15-min increase in walking time [20]. The physical challenges of the path itself will impact walking, with the journey time increasing from changes in elevation, poor or slippery walking surfaces, and traffic [21].This means that studies using simple straight line distances from (or buffers around) water sources [19] will always underestimate effort. The UN recognizes the shortcomings of this definition, and acknowledges that there is an over-estimation of the number of people with water access [22].

Given the spatial complexity in water fetching and the other environmental factors affecting WASH that occur around the household, it is somewhat surprising that more geospatial technologies have not been applied to gain a more detailed understanding of the walk to fetch water [21]. Time taken on a water-fetching path and the impediments faced, have largely been acquired through recall, while many environmental health surveys focus on the immediate home. Few studies have attempted to link the home and the water-fetching path. In many of the environments where water fetching is required, a lack of adequate drainage or sanitation infrastructure means that channels form with general and localized water pooling, a situation that is worsened during flooding, when pathogens can be spread. At lower elevations, mud and water can accumulate, which brings an even greater risk of pathogens. This mud often contains human and animal feces. Households in these low-lying areas or proximate to drainage channels or open sewers are considered to have a higher risk, as do those people who spend disproportionately more time in and around the home, such as the elderly or young children [23]. Yet even those people living in areas with less pathogen risk may still have to walk through riskier areas to fetch water.

Mapping where these challenges occur can reveal negative environmental, social, and behavioral activities that can be targeted through localized interventions, such as explaining why children should not play in and around mud, standing water, or trash, or why vendors should not prepare and sell food near or over grey water and trash [9]. The question is, how can such a map be made?

A few studies have attempted to bring more spatial detail when mapping water accessibility, including using geospatial technologies to better monitor and manage water access in areas with limited resources [24]. Yet these studies identified limitations such as not accounting for topography [19], over-bias toward road networks rather than walking paths [25], global positioning system (GPS) signal errors [26,27], and an inability to distinguish water-fetching walks from other trips [26].

This paper will show a method that can connect both environmental risks around the home and along the water fetching-path while also providing greater spatial and temporal accuracy in the route taken. This study area of Dar es Salaam, Tanzania is a spatial data-challenged environment, with a high disease burden, where access to water requires a high percentage of households having to undertake a walk. However, this is a ubiquitous method that can easily be modified for other locations or other health questions. More specifically, this study challenges the simplistic accessibility distance definition [18], and although it cannot address contextual issues of the source [28], including cleanliness [24,29,30], affordability [31,32], and safety [25], it can provide a more accurate time and distance measurement, and address the physical challenges encountered [21,33], including those which are more normally described in and around the home. The rest of this paper will use the phrases ‘challenges’ as well as ‘risks’ to include impediments to the walk and potential health risks/hazards encountered along the path and in the proximate environment. This study utilizes an on-the-ground geospatial approach to compare multiple water collection routes in frequently researched municipalities of Dar es Salaam, known for their high disease burden. 

## 2. Methods

To more fully and accurately capture a water-fetching path and the environment through which it passes, including challenges that will lengthen the walk, add burden on the fetcher, and pose potential health threats, a technique is required that is easy to use, relatively inexpensive, and suitable for various locations. Spatial video (SV) has been employed in various challenging environments where fine spatial scale data are not available. For example, Curtis and colleagues [34,35] used a car-mounted system to identify water, trash, and animal (as a proxy for rats) patterns in Haiti to identify disease risk and support epidemiological water testing. SV is the combination of a video with attached or embedded GPS coordinates. After collection, key elements are digitized from the video into either Google Earth or a geographic information system (GIS). While vehicle-mounted systems dominate previous research, a few examples have utilized a hand-held approach. This paper is the first time this method has been fully implemented in terms of a systematic walking SV data collection strategy, and in so doing provides a template for other health studies where the walking activity space is the primary focus.

SV provides a rich source of data for the walking path. Water-fetching challenges that have raised concern in the literature, such as how steep or uneven terrain can lengthen walking time, can be located, mapped, and through visual observation quantified in terms of severity. The video component of SV provides a visual way to correct GPS bounce due to the nature of the urban environment, as tightly-packed structures with overhangs impact spatial precision and can even exaggerate the apparent walk length. The video can be used to verify walk purpose and the correct route, two limitations of past geospatial studies [19,25,26,27]. Finally, the walk itself is a transect through the environment, and all the previously described health hazards can be mapped as both challenges to the fetcher and an impression of the risks faced by those living along different sections of the path. 

To illustrate the potential of SV as a research tool to assess variations in water fetching routes, the following questions are posed: Is there geographic variation in the challenges encountered between water fetchers, both within and across study wards? Is there geographic variation in the amount of health risks encountered along the walk? 

Dar es Salaam was chosen for this work for various reasons, not least being that the 2012 Tanzanian census reported that only 33 percent of the city’s households had a piped water connection inside their homes or outside in their yards (see http://www.nbs.go.tz/), and the city’s water supplier estimates that only 20 percent of these connections actually work [28]. A 2015 survey also found that 65 percent of residents believe that access to clean water is the most serious problem in their country [36]. Furthermore, while the city’s population exceeds 4 million, it is estimated that 70 percent live in unplanned informal settlements, which in turn means that there are often severe health problems [37]. 

To map and quantify water fetching routes, subjects were selected from three of Dar es Salaam’s centrally located and oldest residential wards located in the Ilala and Kinondoni municipalities: Buguruni, Ilala, and Magomeni. These wards were chosen since they were established as African neighborhoods during the British colonial era. During this time, colonial policy dictated that African residential areas use public water standpipes rather than receiving the direct home connections found in European residential areas [28]. As a result, few homes in these three areas have direct piped connections, resulting in high incidences of water fetching. Each of these wards is subdivided into smaller sub-units. Subjects were selected from each of these sub-units. Convenience sampling was used since this research was conducted during the day and not all households had someone present to answer questions. The authors of the study sought to select households at varying distances from water sources (i.e., a spatially representative sample). This did not limit video collection to households within the 1 km distance definition of water access since households do not necessarily use the source of water closest to their home [19].

All research participants provided verbal consent after receiving an explanation of the study purposes, its risks, and their rights. This procedure was approved by the researchers’ institutional review board. After providing consent, each subject was followed with a hand-carried SV camera. The camera was a Contour +2, which is a small extreme sports camera with an internal GPS. A rocker bar initiates both video recording and GPS acquisition as soon as it is slid forward, making this suitable field equipment for non-technical collaborators. In the highest definition mode, video is recorded onto a 32 GB micro SD card that can hold approximately 4 h of data. The field researcher allowed the camera to find a satellite signal (a solid green light indicates a fix) before following the water carrier. The camera began with a pan around the water source before the researcher followed behind at approximately 10 meters to record the exact walking path. The camera was held steady to avoid image shake, but the point of view did change if important features were passed, such as trash extending down a side alley or people gathering around a street food vendor.

Research participants were asked to take their normal walking path between their home and their primary water source. Households in Dar es Salaam often use multiple sources. For example, one source might provide drinking water while another might provide water for household uses such as bathing or cleaning. Secondary sources are also used because of the unreliability of water supply in the city. This study was concerned, however, with the most frequently-utilized source. All collected routes are one way, either from the home to the water point or vice versa; the different directions are attributed to where the participant was first identified. The majority of research participants were women, owing to the gendered nature of water collection [38].The male fetchers were either bachelors, or in one case, a worker collecting for his elderly employers. 

**Figure 1:**
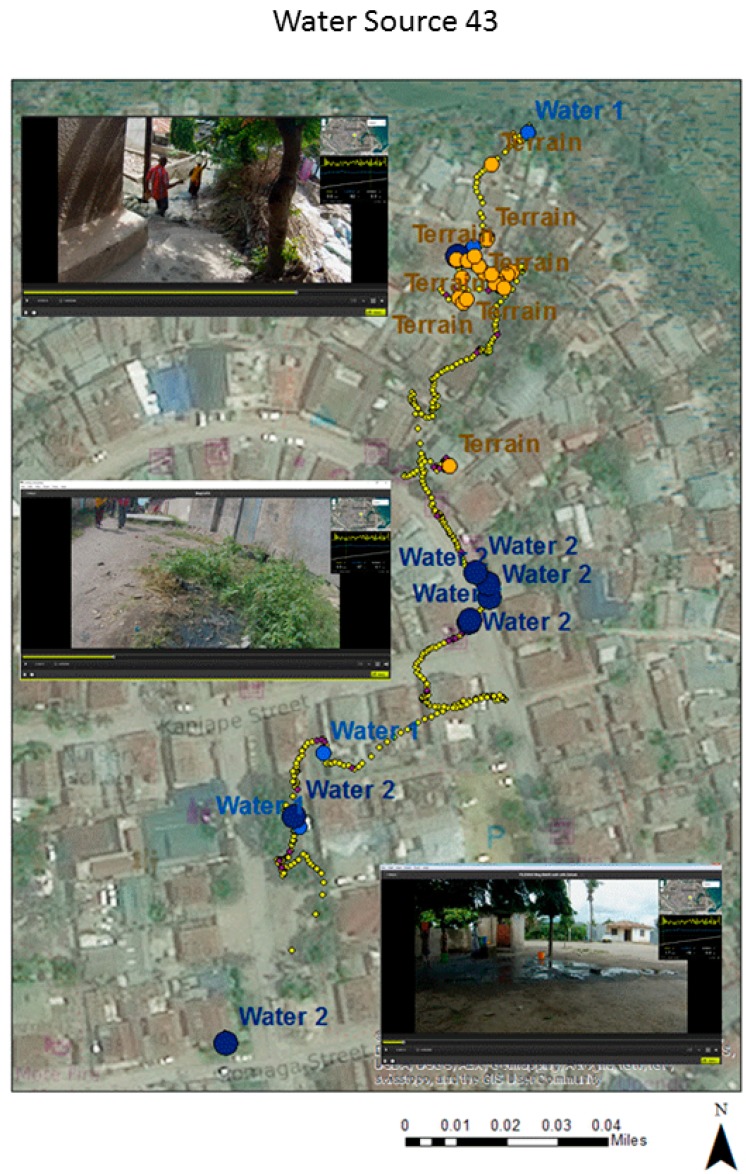
Mapping of challenges along the route of water source 43. The three inset images show the software (Contour Storyteller) used to simultaneously view the video and location. Each image displays a challenge at that location along the path.

**Figure 2:**
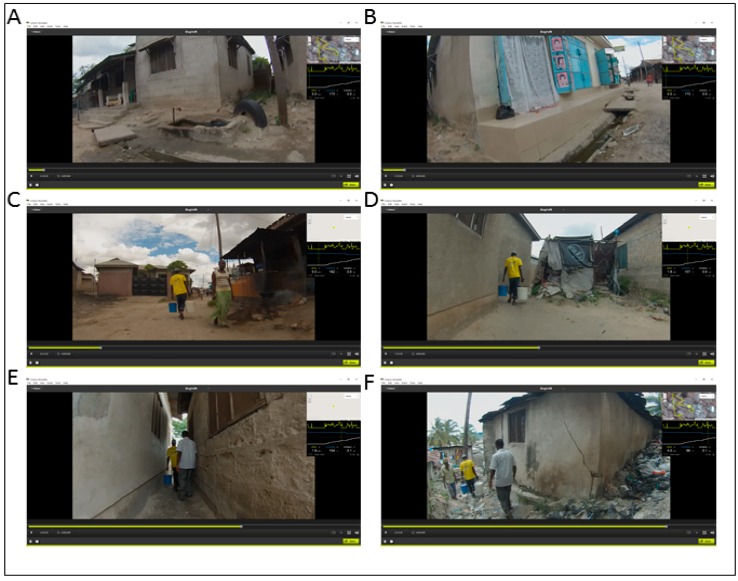
Six example spatial video images showing challenges faced along the walk to water path. Challenges faced by the water carrier include standing water (**A**, **B**), vendors cooking next to the path (**C**), terrain, and trash (**F**). Images **D** and **E** also show how the nature of the built environment results in errors in the GPS path.

At the end of video collection, the GPS path was downloaded using Contour Storyteller, the free viewing software of the camera that allows both video and location to be displayed simultaneously (see the image examples in Figure 1 and Figure 2). Once back in the research lab, each spatial video walk was viewed for challenges pertinent to either the water fetching or informal settlement health research literature. Based on the literature, the following challenges or route context were extracted from the spatial video for subsequent mapping: *Walking challenges* (terrain and traffic), *Contextual variables* (people, children playing, and vendors), and *Health challenges* (trash, standing water, and open drains).

Each route was viewed by a researcher who specializes in the social aspects of water accessibility; in this case, the same researcher that collected the field data. All challenges along the routes and immediately proximate to the fetcher were identified by the SV media time stamp and tabulated for comparative purposes. Following this, a researcher specializing in fine spatial scale health risks in similar environments mapped the challenges identified by the first researcher, and then mapped more general health risks seen anywhere in the video. The second pass also differed in that challenges were digitized at their exact location in Google Earth (which utilizes the same imagery as Contour Storyteller) with the resulting KMZ being imported into a GIS for more flexible mapping. 

## 3. Results

Data were collected over four weeks in June 2014.This video collection was part of a larger water access project, so future studies replicating this method could be done in a shorter timeframe. One day was spent in each of the thirteen ward sub-units described in the methods section. All SV were collected by the same researcher who was accompanied by a research assistant from the University of Dar es Salaam. Thirty one-way water-carrying routes were captured, with an average length of 126.733 s, ranging from just 23 s to 512 s. After the SV were downloaded, each route was viewed by the initial field researcher to identify challenges encountered by the water fetcher, these being matched to the associated SV media time (Table 1). On average, each walk contained almost five different types of challenges and a total of over 33 combined challenges. The nature of the challenges varied between the walks; while the presence of trash and standing water was ubiquitous, the intensity of these varied across the walks. Several other features along the walk pertinent to questions not addressed in this paper, such as the locations of latrines and queues at the water source, were also recorded.

### 3.1. Walking Challenges: Terrain and Traffic

The most common challenge encountered on the routes was difficult, uneven, or steep terrain. For one walk, there were 47 instances of steep terrain; no other challenge appeared as many times in a single video. Examples of such terrain included fetchers climbing or descending staircases or slippery slopes. Most of the stairs also posed hazards as they were visibly crumbling. Therefore, not only did fetchers need to deal with changes in elevation, but the actual paths also appeared unstable. Six walks included continuous stretches of difficult terrain, with one walk including two separate sections. The authors are aware that there is a scale issue when representing a stretch of terrain by single points—and with multiple coders the distance between points (and therefore intensity) may vary. However, all coding was completed by the same person, so there was consistency between walks.

A second challenge that could affect the safety of the fetcher and lengthen the walk was traffic, with eight of the walks being near to cars, motorcycles, and bicycles. Only moving vehicles were coded and only those moving at full speeds; for example, a person pushing a bicycle was not coded. The total incidence of traffic was rather low, being on average less than one vehicle per walk, though in one example there were ten vehicles passing the subject (the water tap was located on the side of the road). 

### 3.2. Contextual Variables: People, Children Playing, and Vendors

Both adults and children were commonly observed on the walks, with an average of over 10 people for each walk (walk 31 had the maximum of 25 people). All the people coded were in immediate proximity to the water fetcher; people on the other side of the street were not counted. These included people passed on roads or paths as well as people congregating around the water tap. For example, in one walk, the tap was located outside of a nursery school, so pupils and teachers were within several feet of the tap. If SV coding was extended to the entire view, then the numbers would rise dramatically.

Vendors appeared in 19 of the 30 walks, with an average being less than two per SV. These vendors sold food and non-food items such as clothing and included both stores and carts. Only those places actively selling were coded. A closed store or empty cart was not coded. For most walks, there were few vendors encountered as these were generally residential streets. In one exception, the walk included eight vendors when the fetcher passed through a commercial block. 

### 3.3. Health Challenges: Trash, Standing Water, and Open Drains

Trash was the second most common challenge occurring in all but two of the walks. Only large and identifiable pieces of trash were coded, such as garbage bags, stacks of debris, or discarded clothes. In all but one walk, these were discreet pieces of trash; the one exception being a continuous 20 s stretch where trash lined the walking path. The trash often contained items of concern such as aerosol spray cans, paint cans, and plastic, the last two of which could provide mosquito breeding places.

Standing water was the third most common challenge, occurring in 26 of the walks. Fetchers passed an average exceeding six standing water locations per walk. This water included both puddles and continuous areas of water. Standing water was differentiated as levels 1 to 3 by its depth as perceived by the researcher. The most common depth of water was level 2, which the coder perceived to be pooling water. The water depth often varied along the walk. It is important to note that these videos were collected during Dar es Salaam’s dry season rather than its rainy season, so this standing water was not the result of precipitation. In some instances, the source was clearly visible—for example, near a water point or surrounding a drain—but in others there was no obvious origin. It is possible that this standing water could have contained sewage. Even level 1 often had a muddy residue that, as previously mentioned, is also considered a health risk, as it indicates where water settles. It was also possible to ascertain where water would flow during the rainy season due to the hydraulic erosion seen on the paths.

Drains were encountered on 17 of the walks, with an average of just over three. These included both roadside open drains as well as drains emptying from homes. As with standing water, drains were coded by level; level 1 was dry, while level 4 meant the drain was full. The most common level encountered was level 1, but four walks had drains with multiple levels.

### 3.4. Mapping the Challenges

The first coding of walk-related challenges was aspatial in nature in terms of recording challenges close to the fetcher by the media time stamp. Mapping was achieved by matching the media time of each identified challenge to its associated GPS GMT time and then using a specially written interpolation routine (G-Code) to assign each word to a coordinate by time stamp [39]. Water source 43 is used to illustrate this approach. These spatialized words were brought into ArcGIS 10.4 as a point layer, meaning a query could be used to produce separate challenge maps, such as where all the standing water was located (see Figure 1). The yellow dots show the walking path, while the exaggerated brown and blue dots are the locations of water and terrain challenges. Three inset images taken from the spatial video show examples of the mapped challenges from the corresponding section of the path. The lower two images show both standing water and mud. The top image provides an example of a physical walking challenge. While general patterns of the walk emerge (it is wetter at the beginning of the walk and the terrain worsens towards the end), there is also some GPS error due to the physical nature of the urban environment.

The benefit of the first approach to create a map of challenges is that a collaborator needs no spatial software expertise as only the SV media times are recorded. A second more spatially-accurate approach to coding the walk-related challenges was based on previous work digitizing health risks from a SV source [34,35]. Water source 31 is used to illustrate this approach. A second researcher, familiar with both spatial video coding for different environments and with a broader perspective of health challenges and their environmental signatures, digitized elements from the SV directly into Google Earth (before translation into ArcGIS 10.4). This second researcher used the literature described at the beginning of the paper and the same list of challenges extracted by the first researcher, but this time supported by high-resolution aerial imagery in both Contour Storyteller and Google Earth, to digitize the exact location of where each challenge was found. For example, large trash piles extending away from the walker’s path were coded not just as a weighted point but as a series of points to show extent and directionality. This produced a more accurate representation than just being matched to the GPS coordinate. In the previous coding example, the event was matched to the GPS path and not to the direction or distance where it was observed. Figure 2 displays six visual examples taken from water source 31, beginning with the starting water tap and ending close to a section of uneven terrain sloping upward that was also near to considerable trash piles. The inset maps and their associated elevation graphs show how the terrain became steeper and more difficult to traverse as the walk neared the ending residence. Open drains and water/muddy residue can be seen at the beginning of the walk.

**Figure 3:**
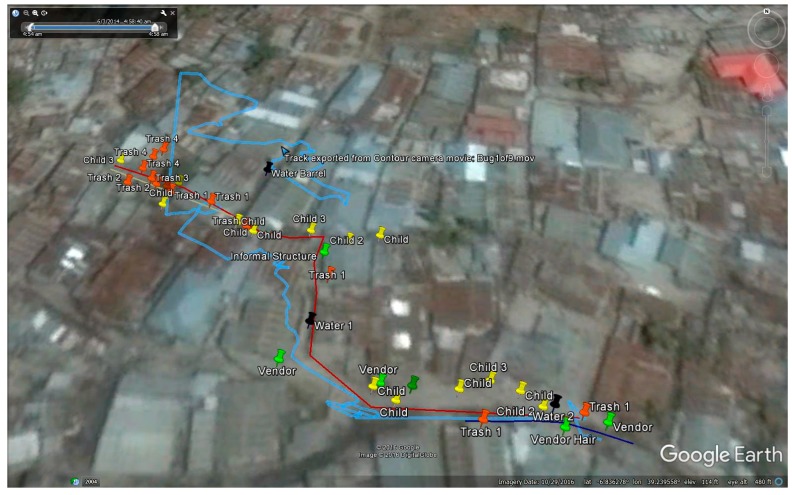
The original GPS path, the route correction, and various digitized risks. Google Earth is preferred over a geographic information system (GIS) due to the imagery available and the flexibility and ease-of-use in digitizing.

Figure 3 displays the original GPS path and the route correction to demonstrate the two steps in the process of digitizing these risks into Google Earth. By following the video while also looking at the GPS points and the overhead imagery, it was possible to ‘correct’ the actual path. While the GPS path is relatively accurate in the initial stages of the walk, considerable drift occurs when the walker enters a section of the built environment with narrow alleys and overhanging roofs (see Figure 2E). The corrected path is marked by pins that are then joined to give an accurate water fetching route. Challenges along this path are digitized at their spatially correct location.

**Figure 4:**
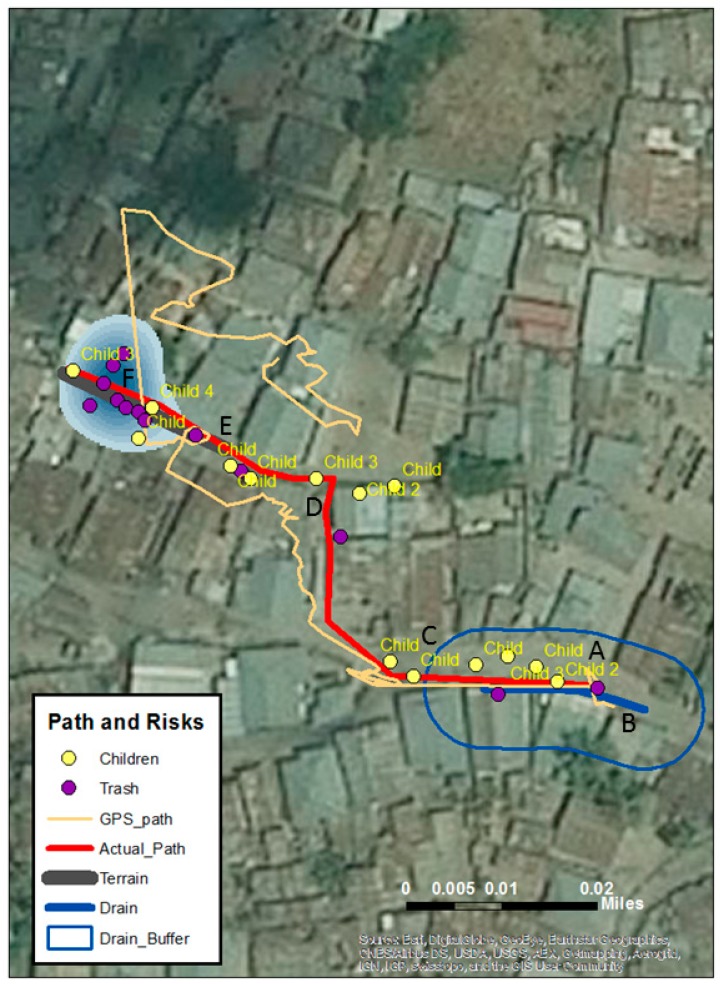
Mapped challenges along a walk to water route that also includes the recorded GPS path and the spatial video corrected path.

Figure 4 displays the final map after translation into ArcGIS 10.4. Just as with Figure 2, two risks have been queried out—in this case, children and trash. A Kernel Density Estimation (KDE) is a commonly used spatial technique in epidemiology to visually show quantity and direction of data concentrations that would otherwise be hard to visualize using point data alone (many of these digitized points had considerable amounts of trash accumulation as can be seen in Figure 2F). KDE has also been used to map out SV digitized points in other projects [35,40]. The original GPS and corrected walking path are also displayed. The water filled drain (Figure 2B) is represented by a line and then buffered for proximate risk which is consistent with Curtis and colleagues [34,35]. 

## 4. Discussion

Access to clean water is a basic human right, but the walk to fetch water remains a daily burden for many households living in areas of economic hardship. These walks can be characterized in a variety of ways: length, time, challenges faced in making the walk (burden on the fetcher), and challenges experienced on or proximate to the walk (burden on the habitable environment). This study focused on water fetchers in Dar es Salaam, an urban area where two-thirds of households do not have piped-on-premises water. While access to clean water is a health imperative for households [4,20], from a spatial perspective it remains an under-researched area.

In addition to the time it takes to fetch water, other important research questions include, where are the likely points of contamination between the water source and the home? What physical challenges does the water fetcher encounter? What are the environmental health risks found along the path? This study used spatial video to address these questions and to quantify and map the challenges, while also providing more spatial (and visual) insight into the daily risk experiences for those who live along these transects in what are traditionally data poor areas. 

While GPS has previously been used to detail water fetching routes [26,27], limitations with that method included knowing the purpose of the walk (for non-researcher attended walks) and adequately accounting for unrelated stops. The accuracy of the GPS signal is also problematic in informal settlements due to the dense concentrations of buildings and narrow alleys. While Figure 1 showed some GPS bounce around a still generally identifiable path, Figure 3 and Figure 4 captured how much error can be involved when the walk goes through narrow corridors with tall or overhanging buildings. This bounce would lead to overestimations in both path and distance measurements if researchers were solely reliant on GPS. However, by using the video, along with reasonably detailed overhead imagery, it was possible to correct this path. This same video also helped verify that no distractions occurred en route.

Table 1 compared challenges that occurred on the walks in the three areas. Walks in Magomeni included the highest average of total challenges (41.167), ranging from a high of 118 to a low of 11. Walks in Magomeni also had the highest average of different challenges (4.917) from a high of 7 to a low of 3. Walks in Ilala had the fewest total challenges and the fewest different challenges. Even so, it had walks with seven different challenges, and all walks in that area had at least 17 total challenges. This amount of heterogeneity in the walks, even for those in the same general area, mirrors studies elsewhere [7], and specifically in Dar es Salaam [37], where conditions associated with malaria prevalence can vary quickly, even within 100 meters.

Future studies could focus on another aspect of heterogeneity concerns when videos are collected. Challenges may vary based on the time of day and season of year. For example, the presence of standing water would likely increase during the rainy season as would the number of full drains. Similarly, more people and more traffic might be encountered in the morning when people are traveling to work or school.

The layout of these neighborhoods means that fetchers are generally walking along two-lane roads or in alleys between homes. In several examples fetchers walked along steep, uneven, and difficult terrain that requires increased exertion and creates the possibility for falls. Other water-fetching challenges were visible on the SV but not mapped: fetchers carrying water on their head, setting water containers down to rest their arms and hands, and walking barefoot (though these occurred with less frequency than anticipated).Unfortunately, the orientation of the camera, with the researcher walking behind the fetcher, was not conducive to capturing any discomfort expressed through facial expressions. However, not all physical impacts would necessarily be visible in a single video. Many of these impacts are cumulative, such as joint pain, arthritis, and caloric deficiencies [41,42]. Furthermore, while these videos show a singular walk, water fetchers make multiple trips each day.The number of trips per day varies, with more trips required on laundry days for example. Even so, SV presents a data source that can be repurposed by different researchers wanting to focus on the physical nature of how water is carried. For example, comparing SV for different countries might reveal differences and similarities between carriers that are difficult to ascertain from more traditional research methods.

Another under-researched area is where potential contamination of the fetched water might occur, such as at the water source, along the route, or at the home. Water quality testing tends to occur either at the point of source or the point of use, so the ability of SV to provide insight into potential contamination during transportation is significant. SV revealed that in many cases, people fetched water in containers without lids, which means that contamination was possible at both the source and along the route. Proximity to traffic is one possible source of contamination. Dar es Salaam’s roads are dusty and traffic can create particulate clouds that can settle in the open containers. Many of the fetchers were near traffic, including using taps located on the side of the road. Also, the containers were placed on the ground at the water source, an area that was often wet and muddy, meaning contamination of the exterior of the vessel could occur. The home environment was not recorded, but future studies could easily extend to show the way water is stored around the home. These insights could be used to either direct educational strategy to change practices or possibly target infrastructure changes, such as placing a large concrete plinth around each water source to reduce mud contamination. The fact that the areas around the water sources themselves were not kept clean offers another area for intervention.

There are many environmental factors found in and around the household that are known to pose a health threat: standing water, mud, open drains, and trash are arguably the most problematic. These have sometimes been surveyed around the home to determine in situ risk, but rarely have these same risks been mapped out along activity paths. It is true that these factors are heterogeneous and dynamic, and each video represents one point in time, but while the specific challenges may vary, their existence persists. The same health and environmental factors observed in these videos would also be applicable to other activity paths such as collecting firewood. Yet the challenges identified in Table 1 were found directly on the route; in some cases, the fetcher had to walk through water or mud. In a city with inadequate sanitation it is possible that the water could contain human waste. Households near to open drainage channels, which may also act as open sewers, and proximate to toilets, especially unimproved pit latrines, also present health risks. Dumping of trash can occur anywhere, especially on unclaimed land, and in some instances quite close to homes, and can again provide mosquito breeding sites and focal points for vermin. Mitigating or exaggerating factors to these challenges include elevation, with higher areas being safer, because water and sewage literally runs downhill. All these factors were visible on the SV and could be mapped as transects of risk through the study areas. If we again consider Figure 1 and Figure 2, both sets of graphs and height readings suggest an approximate 100 foot change in elevation along the route and both contain localized settling areas where water and trash could accumulate. In Figure 2 specifically, the path shows a clear descent toward the end, which produces a greater fetching burden to the individual and also means his home may be vulnerable to runoff, especially during rain events. 

While this detailed mapping can help reveal the risks to the water fetcher, these data can also act as samples of the local environment and further the discussion on the heterogeneity of disease environments in informal areas. The concept of health risk heterogeneity has been well described, if not mapped, at a granular scale. For example, by referring to Figure 2, the end of the route showed a large trash accumulation close to the household. Added to this, the house had no obvious mosquito protection, and children were playing outside. Interventions here could include removing the trash, educating about the dangers of dumping trash in the alley, applying larvicide, providing resources for mosquito-proofing the house, and educating about techniques to avoid bites. While this is a localized intervention, similar strategies (and target locations) could be extracted from all the walks. This is also important from an epidemiological perspective, since targeting only the main ‘hotspots’ may never reduce the overall disease threat in such an heterogeneous environment where localized conditions result in scattered small clusters [37]. While spatial targeting of the main cluster is an effective way to prioritize limited resources, other approaches are needed for the rest of the environment if any breakthrough can occur.

Hotspot maps of mosquito density and malaria prevalence from Mwakalinga and colleagues coincide with most of the water-fetching routes mapped in this paper, especially those in Magomeni. The SV data mapped in this paper offers the opportunity to combine fine-scale environmental settings and behavioral activity that the authors claim is still missing in the research. The authors comment that for effective near-real time intervention (especially for emerging outbreaks), bottom-up community-based strategies [37] are required that can identify (map) these types of risks. It is easy to imagine how an expanded version of the mapped routes we present, especially if coordinated by community groups, could provide invaluable insight to researchers and interventionists. 

Malaria is not the only disease of concern in the informal areas of Dar es Salaam. Cholera has been endemic since 1977, with small outbreaks occurring every year, usually with peaks that coincide with the rainy season. Of real concern is that the biotype found in Dar es Salaam has the potential to cause quick-moving epidemics in dense urban areas [43]. All of the wards in this study are considered at risk for cholera, especially Buguruni since its low-lying areas tend to flood with the rains [44]. All of these wards also appeared in the highest categories of incidence during the 2006 outbreak [8]. 

Cholera’s heterogeneous patterns within the most at-risk wards are due to changes in elevation, accessibility to safe water, inappropriate toilet locations, and inadequate sewage removal [44]. As noted before, it is the poorest areas of the informal settlement where these factors tend to coincide, but previous research on risk lacked the detail needed to tease out the environmental variations that could lead to an outbreak [45,46]. While it is environmental water sources that provide the reservoir needed for cholera’s endemic nature in Dar es Salaam, it is the devastating and explosive human-to-human transmission that is of real concern. In these situations, it is the local conditions, such as small water bodies and the interlinkages between sewage and drainage [47], that help explain what happens within each ward. Environmental, social, and behavioral features gleaned from the video can help target either infrastructure changes or education strategies aimed at those local conditions [48].

Spatial video also offers a possible intervention in terms of the water sources themselves. Although the Tanzania government advocates for universal piped water connections [28], that goal is not realistic in the short-term, so water fetching will continue to be important in Dar es Salaam. SV can provide insight into where to locate these shared water sources to best minimize walking time and health risks from environmental challenges.These location decisions offer an ideal opportunity to involve local communities in the types of public-public partnerships that have already created successful water provision systems in the city [49]. Collaborative community involvement would also consider other environmental or health challenges beyond those discussed here.

There are limitations to this study. First, the coding of the SV relies on researcher perception to determine the depth of water or what constitutes trash. This study attempted standardization by using the same researcher for all coding. It also did not attempt to provide a standard assessment metric (what constitutes a ‘risky’ level of standing water), as each environment is different and should be assessed using local context. For an informal settlement in Dhaka, Bangladesh, there will likely be more overall standing water along a walking route in comparison to the study presented here, but the same approach can be used to characterize that walk and identify context-relevant hotspots. The decision to not present a standardized coding metric is also justified based on the heterogeneity described in this section. Second, the presence of the camera may impact the amount or type of observed challenges. It is possible that the camera might attract attention and therefore increase the number of people observed during a walk to water as people approach the water fetcher. Given the small size of the SV camera and the fact that it is carried discreetly by the researcher, it is unlikely to attract a significant number of bystanders. Regardless of any camera impact, the presence of the camera would not affect other challenges such as standing water, drains, or trash. Third, the camera might influence water-fetching behavior. It is possible that the water fetcher might alter his or her behavior such as taking a different route to a water source. To mitigate that possibility, participants were asked to walk their normal path between water source and home.

It is useful to discuss the logistics of data collection for replication in other locations. While data collection is labor intensive in terms of having to follow the individual, the benefits make this investment worthwhile. The time taken to collect each route is as long as it takes to walk the route. The time taken to process data is a matter of minutes. The time taken to code risks from each route depends on length, and the density of features in the environment, but on average it was two hours per walk. These time investments are negligible, especially if collaborating with a university, given the richness of data which can also be repurposed for other health investigations. A university partner would use students to perform the coding, with the benefit to them being real-world experience, a richer understanding of the problems faced in informal settlements, and the experience of working with this technology. This approach has been extremely successful at Kent State University, where every semester there are multiple international collaborations. Once the local team sees the benefit in this approach, it often becomes part of a broader health strategy. Each camera is relatively inexpensive (approximately $200), all software is free, and the GPS is inbuilt, meaning no additional equipment is needed. 

## 5. Conclusions

The environmental and physical challenges experienced on a water-fetching walk have real and meaningful impacts on water access. It has been shown that thresholds of effort, usually expressed as distance or time, can affect the health of the household. Here we have shown how this effort can be better quantified. All subjects in this study encountered challenges that caused additional impediments to a simple measure of distance (especially a simplistic straight-line distance) and the associated calculation of time spent. Apart from being able to consider the path of the walker and the intent of the walker, this study was also able to eliminate other problems faced in more aggregate studies such as which water source was utilized. 

This study has also shown that SV used to capture the water-fetching walk can be repurposed to provide an insight into the daily health challenges faced by water fetchers along that path. In addition, these paths can be viewed as transects of the neighborhoods, and an indicator of the types of behavioral activities, such as children playing in mud, that can also lead to disease. As a result, the maps not only show the daily risks that may affect the health of the water fetcher, but also provide spatially detailed insights into the broader environment through which he or she walks. In so doing, we generate the type of data so frequently lamented as being missing in many informal settlement health studies. This shows how the heterogeneity of an at-risk and data poor environment can be mapped. It is not hard to imagine how the combination of these and other walks/transects could be used to organically grow a map of health risks for these communities, and help determine intervention strategies through infrastructure design (trash removal or drainage changes) or education. 

## Figures and Tables

**Table 1:** Challenges observed in the walk videos.

Walk	Ward	Walk Length (S)	Total People	Vendors	Trash	Water			Drain				Terrain	Traffic	Total Hazards	Total Different Hazards
						Level 1	Level 2	Level 3	Level 1	Level 2	Level 3	Level 4				
66	Ilala	81	13	0	5	3	11	0	0	32	3	0	0	0	32	3
65b	Ilala	77	9	1	4	1	2	0	0	17	4	0	0	0	17	4
65a	Ilala	55	11	0	2	2	2	0	0	17	3	0	0	0	17	3
64	Ilala	117	15	2	3	1	4	0	0	25	4	0	0	0	25	4
63	Ilala	111	9	0	3	1	1	1	1	20	6	3	1	1	20	6
62	Ilala	176	6	2	9	1	1	12	10	50	7	0	12	10	50	7
61	Ilala	75	8	1	4	0	1	1	8	34	7	0	1	8	34	7
59	Ilala	78	4	0	1	0	0	21	0	28	4	0	21	0	28	4
57	Ilala	119	11	2	0	1	1	5	0	20	4	0	5	0	20	4
56	Magomeni	94	7	1	0	0	0	13	0	23	4	0	13	0	23	4
55	Magomeni	57	0	0	3	1	0	3	0	11	4	0	3	0	11	4
54	Magomeni	71	8	3	3	0	0	1	2	18	6	0	1	2	18	6
52	Magomeni	42	2	1	6	3	0	0	0	27	5	0	0	0	27	5
51	Magomeni	115	6	0	7	3	1	7	0	25	5	0	7	0	25	5
50	Magomeni	216	16	8	17	2	2	38	0	85	6	0	38	0	85	6
48	Magomeni	123	12	0	2	2	0	32	0	48	4	0	32	0	48	4
47	Magomeni	125	9	3	7	9	16	0	0	54	4	0	0	0	54	4
46	Magomeni	101	24	1	6	2	2	0	1	36	5	0	0	1	36	5
45	Magomeni	265	17	2	4	3	7	1	1	36	7	0	1	1	36	7
44	Magomeni	259	8	0	1	3	1	0	0	13	3	0	0	0	13	3
43	Magomeni	512	16	5	6	26	16	47	0	118	6	0	47	0	118	6
41	Buguruni	100	6	1	15	1	0	0	0	23	4	0	0	0	23	4
40	Buguruni	105	12	2	4	4	0	3	0	25	5	0	3	0	25	5
38	Buguruni	80	22	1	3	0	3	0	1	31	6	0	0	1	31	6
37	Buguruni	46	6	2	1	4	1	0	0	30	5	0	0	0	30	5
36	Buguruni	23	9	3	3	1	2	0	1	19	5	0	0	1	19	5
35	Buguruni	284	18	0	9	7	11	2	0	48	5	0	2	0	48	5
34	Buguruni	44	1	0	3	0	0	1	0	6	4	0	1	0	6	4
33	Buguruni	37	1	0	4	2	2	0	0	9	3	0	0	0	9	3
31	Buguruni	214	25	3	8	4	1	22	0	82	6	0	22	0	82	6
